# Liver stiffness measurement and spleen diameter as predictors for the presence of esophageal varices in chronic hepatitis C patients

**DOI:** 10.1097/MD.0000000000008621

**Published:** 2017-11-17

**Authors:** Mohammed Tag-Adeen, Mohamed Alsenbesy, Ali Abdelrahman Ghweil, M. Ali Hussein Abd Elrazek, Elsayed A. Elgohary, Mohammad M. Sallam, Ali Ismael, Abdallah Nawara

**Affiliations:** a Department of Internal Medicine, Qena School of Medicine, South Valley University, Qena; b Department of Tropical Medicine and Gastroenterology, Qena School of Medicine, South Valley University, Qena; c Department of Tropical Medicine and Gastroenterology, Aswan School of Medicine, Aswan University, Aswan; d Department of Internal Medicine, Zagazig School of Medicine, Zagazig University, As-Sharqia, Egypt.

**Keywords:** data mining, esophageal varices, Fibroscan, splenic diameter, Ttransient elastography

## Abstract

Although it is an invasive and unpleasant procedure, esophagogastroduodenoscopy (EGD) is still the gold standard for esophageal varices (EV) detection. The aim of this study was to investigate liver stiffness measurement (LSM) and spleen diameter as simple noninvasive tools for EV prediction in chronic hepatitis C patients (CHC).

A total of 123 Egyptian patients with CHC have been included and were classified based on screening EGD result into 2 groups; group A (without EV) and group B (with EV). Group (B) was subclassified according to EV grade into 4 subgroups: (B1, grade I), (B2, grade II), (B3, grade III), and (B4, grade IV). LSM was taken for each patient on the next day by an independent Fibroscan operator and correlated to the EGD result. Demographic, clinical, and biochemical data were recorded and analyzed using advanced data-mining computational technology.

Mean LSM was 9.94 ± 6 kPa for group A and 33.32 ± 14 kPa for group B, whereas it was 21.22 ± 3, 25.72 ± 6, 33.82 ± 8, and 46.1 ± 15 kPa for subgroups B1, B2, B3, and B4, respectively. Mean spleen diameter was 11.09 ± 1.7 cm for group A and 16.58 ± 1.6 cm for group B. However, LSM ≥17 kPa was the only independent factor for EV prediction; splenic longitudinal span ≥15 cm was a complementary predictor when LSM was <17 kPa. The overall accuracy was 98.33 ± 3.33, Mikro = 98.26%.

LSM ≥17 kPa and spleen diameter ≥15 cm is a simple noninvasive algorithm that could be used for prediction of EV and discrimination among its different grades.

## 1 Introduction

Hepatitis C virus (HCV) is a worldwide health problem and a leading cause of chronic liver disease,^[1]^ with the highest HCV prevalence being reported in Egypt.^[2,3]^ Liver fibrosis is the major consequence of chronic hepatitis C (CHC) and representing a major global health problem.^[4]^

Approximately half of patients with cirrhosis have esophageal varices (EV), and one-third of all patients with varices will develop variceal hemorrhage, a major cause of morbidity and mortality in cirrhotics. Esophagogastroduodenoscopy (EGD) is the gold standard for EV detection, but a generalized program of periodical EGD in patients with chronic liver disease might result in a heavy economic burden even for developed countries. Furthermore, repeated examinations when not performed under profound sedation are often poorly accepted by patients who may refuse further follow-up.^[5–7]^

Although the overall survival has steadily improved over the last 40 years, mortality following variceal rupture is still closely related to failure to control bleeding or early rebleeding and this is not uncommon during the first days to 6 weeks after admission.^[8–11]^ As consequence of high prevalence of HCV and schistosomiasis, Egypt has a large burden of chronic liver disease, and despite the advent of endoscopy and endoscopic therapy, access to medical centers with experienced medical staff and adequate equipment in Egypt is still limited.^[12]^

Transient elastography (TE) is a noninvasive, ultrasound technique-based technology that assesses liver stiffness measurement (LSM). Established evidences indicated that TE has good sensitivity and specificity for fibrosis, significant fibrosis, and cirrhosis, and it became popular over the last few years.^[13,14]^ Recently, a good correlation between LSM and the presence of portal hypertension (PHT) and EV has been reported, suggesting that LSM could be an interesting tool for predicting the presence of large EV and selecting patients for endoscopic screening.^[14–17]^

Using data mining in applied medicine is important to predict factors lead to disease progression or regression in an intelligent technology fashion.^[18]^ The aim of this study was to investigate LSM and spleen diameter as simple, cheap and non-invasive tools for prediction of EV in CHC patients.

## 2 Patients and methods

From the January 1, 2016 to August 1, 2016, 123 consecutive CHC Egyptian patients have been enrolled in the study. All patients have attended the endoscopy unit of Qena University Hospital for screening EGD before DAAs therapy. CHC had been diagnosed by HCV ELIZA Ab and confirmed by HCV RNA-PCR tests. Full history taking, clinical examination, and significant laboratory findings, including CBC, HBsAg, anti-bilharzial Ab, ALT, AST, PT, PC, INR, serum albumin, serum bilirubin, FBS, and serum creatinine, were recorded.

Based on the EGD result, patients have been classified into 2 main groups: no EV group (group A, n = 60) and EV group (group B, n = 63) which then was further subclassified according to EV grade into 4 subgroups: B1 (grade I EV; n = 14), B2 (grade II EV; n = 14), B3 (grade III EV; n = 14), and B4 (grade IV EV; n = 21). Abdominal ultrasound (US) and transient elastography (TE) were performed for all selected patients after 8-hour of fasting in the day next to EGD.

### 2.1 Esophagogastroduodenoscopy

EGD was done using Pentax EG-2990i Gastroscope (Pentax Medical, HOYA Corporation, Tokyo, Japan) by a single expert endoscopist blinded for the detailed clinical and laboratory data of the patients. EV were classified according to modified Thakeb classification^[19,20]^ as follows:

- Grade 1: Small straight cords of varices confined to the lower third of esophagus.
- Grade 2: Moderate-sized clubbed varices confined to the lower half of the esophagus, with well-defined areas of normal mucosa in-between.
- Grade 3: Gross varices extending into the upper half of the esophagus, with dilated capillaries in-between and normal mucosa might not be visible unless the esophagus is fully distended with air.
- Grade 4: Gross varices extending into the upper half of the esophagus with dilated capillaries on top or in-between and encroaching on esophageal lumen.

### 2.2 Abdominal ultrasound

Using convex ultrasound probe 3 to 5 μHz (Toshiba nemio MX, Toshiba Medical Corporation, Tokyo, Japan), spleen has been measured in the longitudinal plane putting its hilum at the center of the image with recording of the maximum splenic diameter.

### 2.3 Transient elastography and controlled attenuation parameter

Both TE and CAP were obtained using FibroScan device (FibroScan, Echosens, France) by an expert FibroScan operator who was blinded about patient data, US, and EGD results. LSM was performed in the right lobe of the liver through the intercostal spaces while the patient in the supine position. Appropriate probe, either M or XL, was chosen automatically by the device's default based on the amount of subcutaneous fat and skin-liver capsule distance, and accurate probe positioning was confirmed using liver targeting tool with checking all green indicators before pressing the probe button. Result has not been considered reliable except after acquisition of 12 successful readings, with IQR/median ratio <30% and a success rate not <60%.

### 2.4 Exclusion criteria

Patients with BMI >35 kg/m^2^, ascites, HCC, history of schistosomiasis or alcohol intake, HBV or HIV coinfections, and those who received any medications or procedures that could affect portal pressure or degree of varices such as beta blockers, transjugular intrahepatic portosystemic shunt (TIPSS), endoscopic variceal band ligation (EBL), or endoscopic injection sclerotherapy (EIS).

### 2.5 Statistical analyses

All statistical analyses were performed using SPSS version 22 software for Microsoft Windows (Statistical Package for the Social Sciences; SPSS Inc., Chicago, IL). The descriptive data were summarized as frequencies, percentages, and mean with standard deviations (SD). Chi-square test was applied for testing relationships on categorical variables. Differences were considered statistically significant when *P* < .01. The model discriminatory ability was verified through the operational characteristic curve. Calibration of the model by the Hosmer–Lemeshow test showed no significance (*P* = 1.000), indicating that the model was correctly calibrated.

### 2.6 Data-mining analysis

Data-mining analysis is a process by which a computer examines a certain data to create an algorithm. Both Naïve Baÿes (10-fold cross-validation) and a decision-tree model were used. The descriptive Rapid I models of Rapid Miner Program, ver 4.6 (Germany) were initially generated to determine the most significant independent variable in each stage of predicting dependent variables using the computational analysis superior to traditional statistical analysis.

## 3 Results

This study was conducted among 123 CHC Egyptian patients, 78 males (63.4%) and 45 females (36.6%), age range: 28 to 77 years, all patients were subjected to screening EGD before DAAs therapy. Group A (no EV group) included 60/123 patients (48.78%), whereas group B (EV group) included 63/123 patients (51.22%). Patients in group B were subclassified according to EV grades into B1: grade I EV (n = 14/63; 22.22%), B2: grade II EV (n = 14/63; 22.22%), B3: grade III EV (n = 14/63; 22.22%) and B4: grade IV EV (n = 21/63; 33.34%).

Mean age was 51.78 ± 9.6 in group A and 55.28 ± 7 in group B, and 53.71 ± 7, 55.43 ± 7, 55.14 ± 9, and 56.33 ± 5 years in subgroups B1, B2, B3, and B4, respectively. Mean BMI was 29.58 ± 2.3 for group A and 29.76 ± 2.8 for group B, and 28.85 ± 3, 29.35 ± 3, 30.92 ± 3, and 29.85 ± 2 kg/m^2^ for subgroups B1, B2, B3, and B4, respectively.

**Figure 1:**
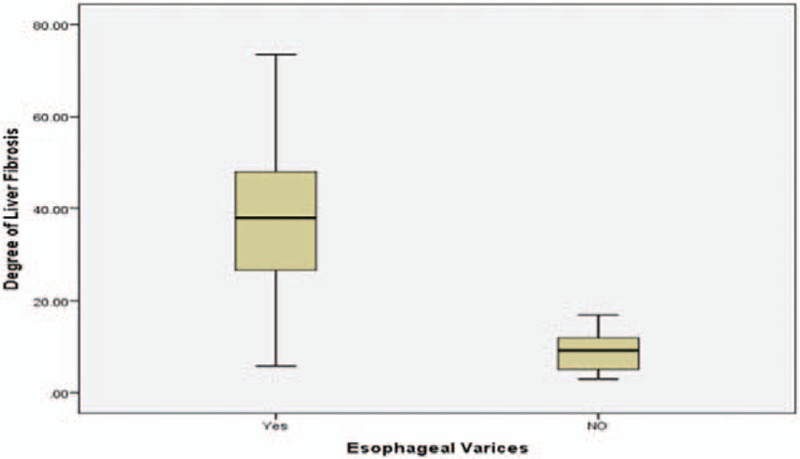
Histogram represents liver stiffness by Fibroscan in group B collectively and group A.

**Figure 2:**
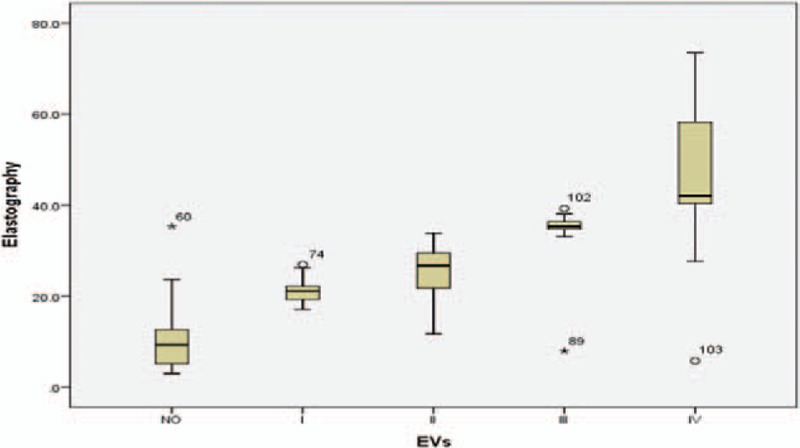
Histogram represents the relation between liver stiffness by Fibroscan and esophageal varices.

**Figure 3:**
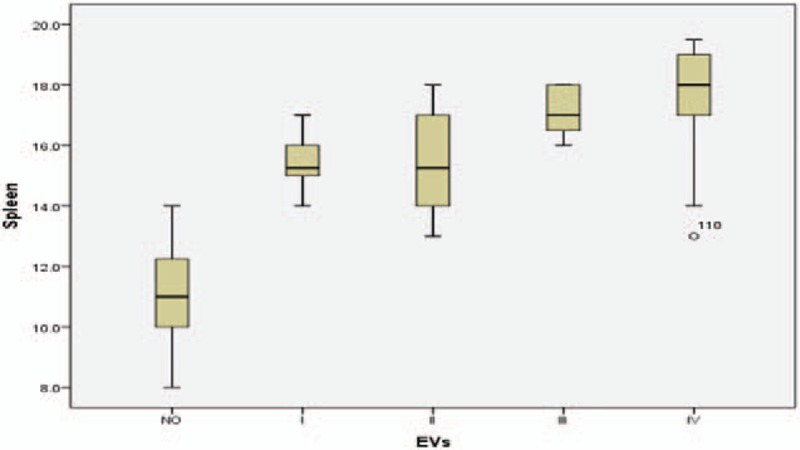
Histogram represents the relation between splenic diameter by US and esophageal varices.

Mean LSM was 9.94 ± 6 kPa (range: 3–35.3) in group A, 33.32 ± 14 kPa (range: 5.8–73.5) in group B, and 21.22 ± 3 (range: 17.1–27), 25.72 ± 6 (range: 11.7–33.8), 33.82 ± 8 (range: 8–39.2), and 46.1 ± 15 kPa (range: 5.8–73.5) in subgroups B1, B2, B3, and B4, respectively. Mean spleen diameter was in group A: 11.09 ± 1.7 cm (rang: 8–14), in group B: 16.58 ± 1.6 cm (range: 13–19.5), whereas in subgroups B1, B2, B3, and B4 it was 15.50 (range: 14–17), 15.50 (range: 13–18), 17.14 (range: 16–18), 17.64 cm (range: 13–19.5), respectively (Tables 1 and 2 and Figs. 1–3).

**Table 1:**
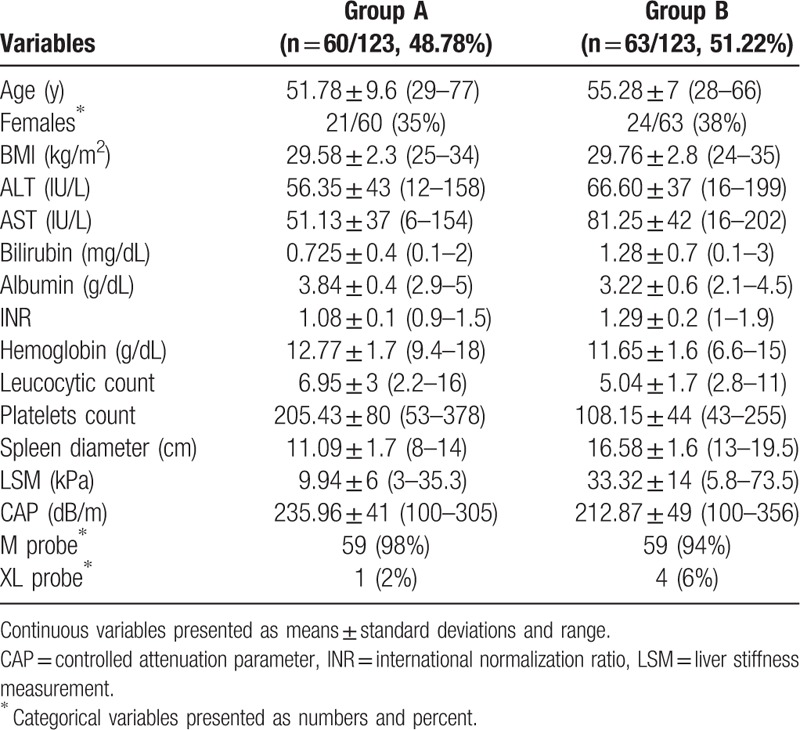
Distribution of different baseline variables between group A (patients without EV) and group B (patients with EV).

**Table 2:**
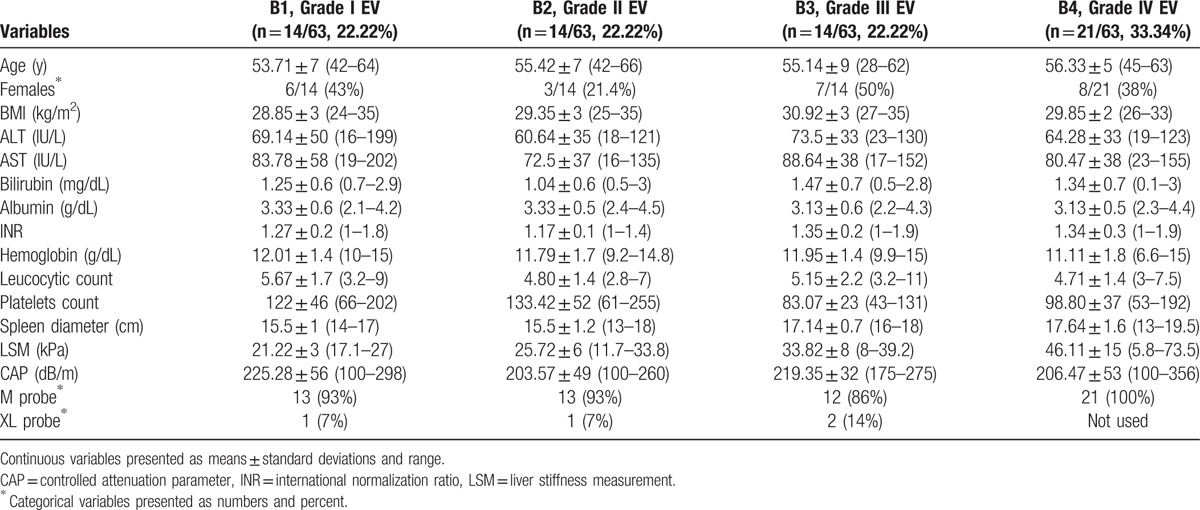
Distribution of different variables among B subgroups.

**Figure 4:**
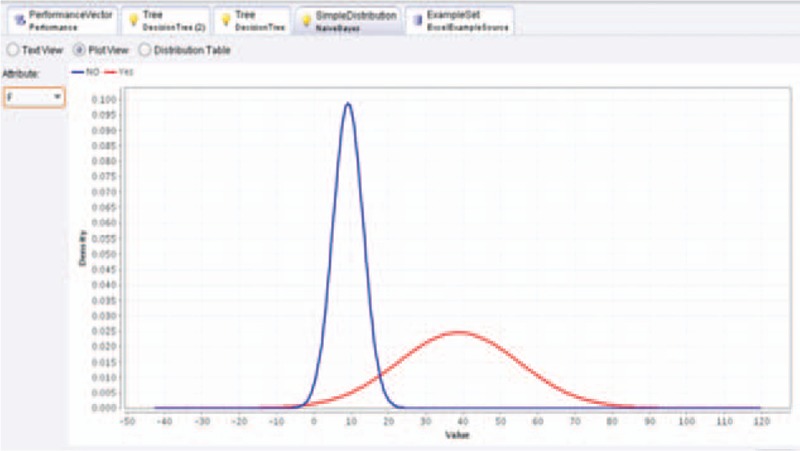
Liver stiffness would be the independent factor for prediction of esophageal varices using Naïve Bayes analysis.

**Figure 5:**
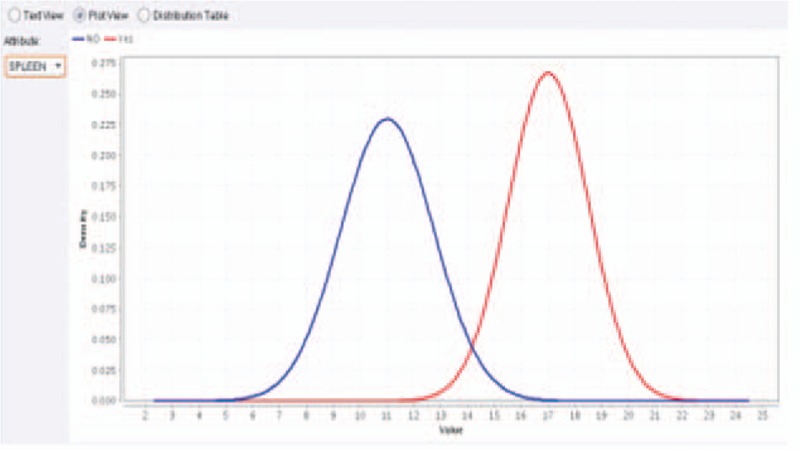
Splenic diameter should be the predictor for esophageal varices when liver stiffness <17 kPa using Naïve Bayes analysis.

**Figure 6:**
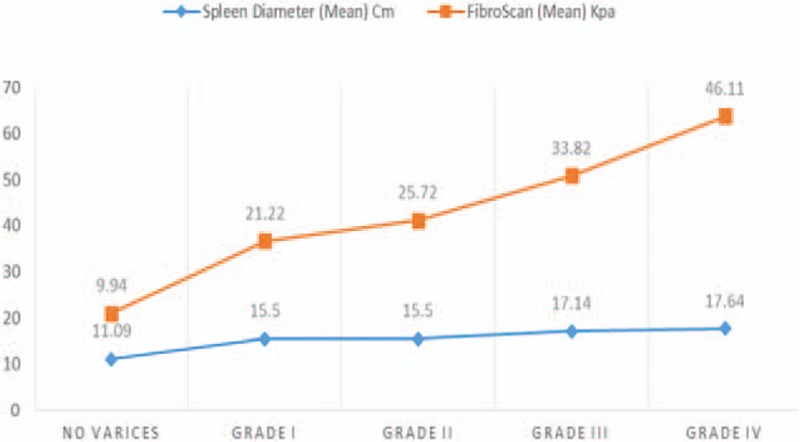
Correlation between liver stiffness and splenic diameter with the presence of esophageal varices.

**Figure 7:**
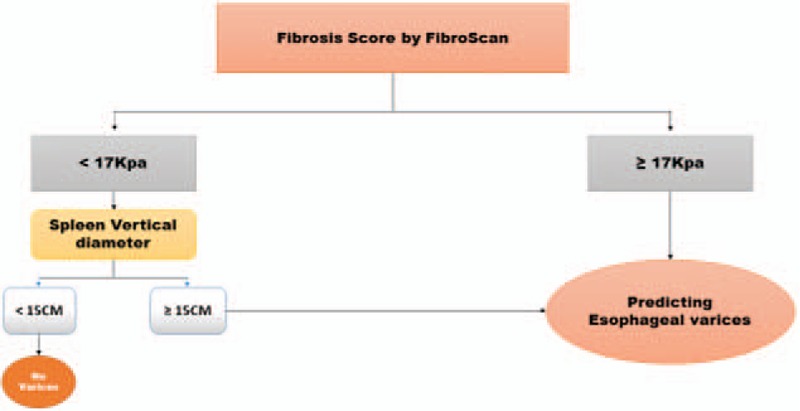
Modified algorithm of decision tree created by Rapid I ver.4.6.

As shown in Table 3 and Figures 4 to 7, LSM ≥17 kPa was the only independent factor for the prediction of EV in the studied patients. However, splenic longitudinal span ≥15 cm was another predictor when LSM was <17 kPa. Decision tree and Naïve Bayes showed significant correlations, Mikro = 98.26%. Accuracy 98.33 ± 3.33.

**Table 3:**
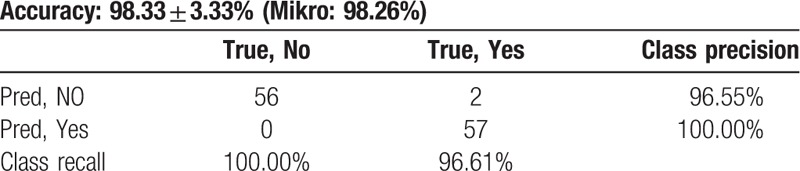
Accuracy of the correlation between liver stiffness and splenic diameter to esophageal varices by Rapid I ver.4.6, Mikro = 98.26%.

## 4 Discussion

Portal hypertension is the hemodynamic abnormality associated with the most severe complications of cirrhosis, including ascites, hepatic encephalopathy, and bleeding from gastroesophageal varices. Variceal bleeding is a medical emergency associated with a mortality that, in spite of recent progress, is still in the order of 10% to 20% at 6 weeks.^[21]^

The American Association for the Study of Liver Disease^[22]^ and the Baveno VI consensus conference^[21]^ recommend endoscopic screening for cirrhotics as a primary preventive measure for variceal bleeding which might place a heavy burden on endoscopy units and cause a detrimental effect on patient compliance. However, patients with an LSM <20 kPa and a platelet count >150,000 have a very low risk of having varices and they can avoid screening endoscopy.^[21]^

Consequently, the search for a noninvasive tool to predict the presence of EVs has encouraged the development of various algorithms based on laboratory parameters, ultrasonography, LSM, spleen stiffness, and spleen size, alone or in combination.^[23–29]^ Aiming to develop a simple algorithm for prediction of EV using noninvasive tools, we conducted this study among 123 CHC Egyptian patients, with no racial or socioeconomic differences. In addition, there were no statistically significant differences regarding age and BMI between groups A and B. As genotype 4 is the most prevalent HCV genotype in Egypt^[30–32]^ and TE is disease specific with different HCV genotypes can generate different elastographic cutoff readings,^[33–35]^ our study was limited to genotype 4 while patients with other liver diseases such as chronic HBV, HCC, alcoholic, and autoimmune hepatitis were excluded. Also, schistosomiasis was excluded based on history, antischistosomal antibody negativity, and the absence of significant periportal fibrosis in ultrasound examination.^[36]^

The mean LSM was 9.94 ± 6 kPa in group A and 33.32 ± 6 kPa in group B (*P* < .0001), this result agreed with many previous studies which have showed that LSM is a useful tool for prediction of the presence of EV.^[37–43]^ On the contrary, the mean LSM was 21.22 ± 3, 25.72 ± 6, 33.82 ± 8, and 46.11 ± 15 kPa in subgroups B1, B2, B3, and B4 respectively (*P* < .0001) which highlighted the efficacy of LSM not only in prediction of the presence of EV but also in differentiation among its different grades.^[37–43]^ LSM cutoff value ≥17 kPa was a good predictor for the presence of EV with 93.6% sensitivity, 95% specificity, 95.1% PPV, and 93.4% NPV in our result; however, previous studies showed different cutoff values.^[41–46]^

Although it usually correlates with the degree of PHT, many studies have reported that LSM should not be used alone to diagnose the presence of EV.^[24,42–48]^ Accordingly, measurement of spleen diameter in the present study was helpful as a complementary tool for EV prediction as indicated by significantly higher mean spleen diameter in group B than in group A (16.58 ± 1.6 vs 11.09 ± 1.7, respectively; *P* < .0001). But the difference was statistically insignificant among B subgroups (15.5 ± 1, 15.5 ± 1.2, 17.1 ± 0.7, and 17.6 ± 1.6 for subgroups B1, B2, B3, and B4, respectively; *P* = .01) which made it difficult to use spleen diameter alone to discriminate among the different grades of EV.

Rapid I, ver.4.6, Berlin, Germany has showed that LSM and spleen longitudinal span were the independent variables which could predict the presence of EV in the studied patients. Using cutoff values of 17 kPa for LSM and 15 cm for spleen diameter, the accuracy of the model for prediction of the presence of EV was 98.33 ± 3.33, Mikro = 98.26% (Table 3; Fig. 7).

The use of data mining in applied medicine is important to predict factors leading to much innovation and heavy creation by extracting hidden factors have never been watched or minded before by traditional statistical methods.^[49,50]^ In this study, a 10-fold cross-validation using Naïve Bayes applications was used to predict the performance of a model on a validation set using computation in place of mathematical analysis.

The main limitation of our study was the inclusion of a small number of patients with the same etiology for liver disease (CHC). Also, despite TE was accurate for detecting clinically significant PHT with a mean area under the receiver-operating curve (AUROC) of 0.93 in a recent meta-analysis,^[51]^ it has certain limitations in ascetic and obese patients.^[35]^ Furthermore, most of available data about its use in PHT detection have been obtained in patients with untreated viral cirrhosis and alcoholic cirrhosis, whereas data regarding other etiologies and data in patients who have eliminated HCV require further investigation.^[22]^ However, newer methods like point shear-wave elastography, 2-dimensional real-time shear-wave elastography, and magnetic resonance elastography show promising results^[47,52–55]^ and could represent future research points.

In conclusion, the combination of LSM ≥17 kPa and spleen diameter ≥15 cm is a simple algorithm that could be used in clinical practice as a noninvasive tool for prediction of EV and discrimination among its different grades in CHC patients.
